# Hydrogen Sulfide—Clues from Evolution and Implication for Neonatal Respiratory Diseases

**DOI:** 10.3390/children8030213

**Published:** 2021-03-11

**Authors:** Abhrajit Ganguly, Gaston Ofman, Peter F Vitiello

**Affiliations:** Center for Pregnancy and Newborn Research, Department of Pediatrics, Section of Neonatal-Perinatal Medicine, University of Oklahoma Health Sciences Center, Oklahoma City, OK 73104, USA; gaston-ofman@ouhsc.edu (G.O.); peter-vitiello@ouhsc.edu (P.F.V.)

**Keywords:** hydrogen sulfide, bronchopulmonary dysplasia, prematurity, neonatal lung diseases

## Abstract

Reactive oxygen species (ROS) have been the focus of redox research in the realm of oxidative neonatal respiratory diseases such as bronchopulmonary dysplasia (BPD). Over the years, nitric oxide (NO) and carbon monoxide (CO) have been identified as important gaseous signaling molecules involved in modulating the redox homeostasis in the developing lung. While animal data targeting aspects of these redox pathways have been promising in treating and/or preventing experimental models of neonatal lung disease, none are particularly effective in human neonatal clinical trials. In recent years, hydrogen sulfide (H_2_S) has emerged as a novel gasotransmitter involved in a magnitude of cellular signaling pathways and functions. The importance of H_2_S signaling may lie in the fact that early life-forms evolved in a nearly anoxic, sulfur-rich environment and were dependent on H_2_S for energy. Recent studies have demonstrated an important role of H_2_S and its synthesizing enzymes in lung development, which normally takes place in a relatively hypoxic intrauterine environment. In this review, we look at clues from evolution and explore the important role that the H_2_S signaling pathway may play in oxidative neonatal respiratory diseases and discuss future opportunities to explore this phenomenon in the context of neonatal chronic lung disease.

## 1. Introduction

Organisms function in a tightly balanced redox environment influenced by the reactivities of oxidants and antioxidants. At steady state, slightly more oxidants (termed ‘oxidative eustress’) are necessary for critical cellular processes to occur [1]. If, however, the balance is shifted further towards oxidants, the phenomenon is termed ‘oxidative stress’ and can trigger an array of signaling and compensatory mechanisms [2]. Babies who are born prematurely encounter oxidative stress in several forms [3]. Under normal circumstances, the human fetus develops in a relatively hypoxic environment in the womb when compared to the outside world. The sudden increase in the partial pressure of oxygen presents an uphill battle against the detrimental effects of reactive oxygen species (ROS) [4]. Additionally, ROS can develop secondarily to infection, inflammation, and reperfusion. Several antioxidant mechanisms are active in the lungs of premature babies to counteract the effects of oxidants, which include the glutathione (GSH) and thioredoxin (Trx) systems, superoxide dismutase (SOD), and catalase, among others. Many of these antioxidant systems develop in a similar timeline to that of pulmonary surfactants, which means that they are underdeveloped in prematurely born babies, making them further vulnerable to oxidative stress-related damage [5]. While the physiological influence of these redox perturbations has been well documented in premature babies, no single antioxidant treatment has been proven to be particularly effective in treating or preventing neonatal respiratory diseases [6]. While antioxidant specificity and tissue delivery have been major hurdles that we have yet to overcome, the current situation also raises the possibility that perhaps regulation of redox homeostasis in the premature and developing lung requires further exploration.

Recent advances in H_2_S research have shed some light on the anti-inflammatory, antiapoptotic, antioxidant, and other potential beneficial effects of H_2_S [7]. Furthermore, H_2_S and its synthesizing enzymes have been shown to play an important role in lung and airway development [8,9,10], thus raising the question: ‘Is H_2_S the missing link in the lung redox homeostasis?’. To answer this intriguing question, we will have to look at evolutionary clues and understand the context in which these various redox systems developed and evolved under different oxidative environments. In this review, we will attempt to address this overarching question and hopefully also raise novel questions in the context of oxidative neonatal respiratory diseases with a focus on bronchopulmonary dysplasia (BPD).

## 2. Evolution of Oxidant and Antioxidant Pathways and the Importance of H_2_S

A vast majority of organisms now depend on the sun for their energy. Plants and many bacteria use oxygenic photosynthesis to transfer electrons from water to carbon dioxide, generating oxygen as a byproduct [11]. Aerobic organisms essentially reverse these chemical reactions in aerobic metabolism. It is interesting to note that millions of years ago, when life first started on this planet, the atmosphere was nearly anoxic. Consequently, oxygenic photosynthesis was not the predominant source of energy for early life-forms. In this context, the sulfur cycle and hydrogen sulfide were essential for the survival of these species. The remnants of this evolutionary process persist in anaerobic microorganisms such as sulfate-reducing bacteria that can breathe without oxygen [12,13]. These bacteria can survive in extreme and anoxic environments such as near volcanos or hydrothermal vents because of their unique ability to use sulfate as their terminal electron acceptor instead of oxygen. Cyanobacteria were the first organisms to demonstrate oxygenic photosynthesis through four-electron oxidation of two water molecules [14]. As cyanobacteria and plants synthesized more oxygen, oxygen levels slowly rose over millions of years (termed the ‘Great Oxidation Event’) to levels that are comparable to today [15,16]. During this time, there was a paradigm shift from sulfide being a major source of energy to oxygenic photosynthesis becoming the principal source. However, this significant increase in oxygen also posed an existential threat to the extant organisms. Those who were able to evolve and develop antioxidant systems to counter ROS had a significant survival advantage in this new environment.

**Figure 1:**
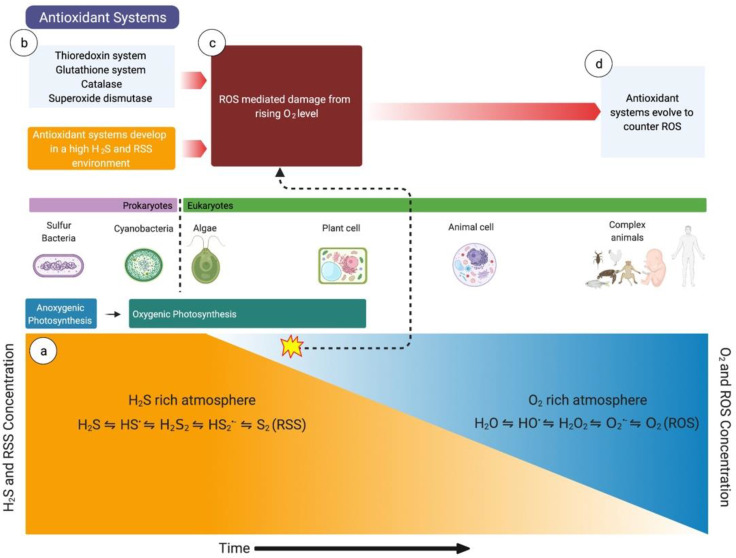
The importance of hydrogen sulfide (H_2_S) in evolution. (**a**) Life started in an atmosphere rich in H_2_S and reactive sulfur species (RSS), where H_2_S was used to convert carbon dioxide to water. (**b**) The antioxidant systems (e.g., glutathione system, thioredoxin system) also developed in H_2_S- and RSS-rich environments. The antioxidant systems initially developed to counter RSS-mediated damage. (**c**) As the oxygen level slowly rose (the ‘Great Oxidation Event’ secondary to oxygenic photosynthesis by cyanobacteria and plants), the extant species were at increased risk of extinction from reactive oxygen species (ROS)-mediated damage. (**d**) To counter the increased ROS-mediated damage, the antioxidant systems evolved over the years to neutralize ROS. HS^•^, thiyl radical; H_2_S_2_, hydrogen persulfide; HS_2_^•−^, supersulfide; S_2_, elemental sulfur; H_2_O, water; HO^•^, hydroxyl radical; H_2_O_2_, hydrogen peroxide; O_2_^•−^, superoxide, O_2_, oxygen.

One school of thought is that a vast number of organisms were able to develop antioxidant systems to counter ROS because they already had enzyme systems to counter reactive sulfur species (RSS) [11]. RSS are intermediary molecules produced through the stepwise one-electron oxidation of H_2_S producing thiyl (HS^•^) and supersulfide (HS_2_^•−^) radicals, hydrogen persulfide (H_2_S_2_), and elemental sulfur [17]. While there are several ROS that have been described, hydrogen peroxide (H_2_O_2_) is generally believed as the principal ROS signaling molecule as it is more stable than superoxide (O_2_^•−^) or the hydroxyl radical (HO·), which rapidly dissolves into peroxide and water [18,19]. H_2_O_2_, however, is promptly scavenged once produced by cellular antioxidant systems [18]. There are many aspects of ROS and RSS metabolism that are similar, but RSS signaling is generally considered more versatile [7,20]. A possible reason for this is that H_2_S signaling and RSS are ancient, and most extant organisms evolved to develop enzyme systems to scavenge them and eventually use them as important signaling molecules [21]. As atmospheric levels of oxygen rose (and the H_2_S level decreased) and ROS-mediated damage posed a considerable existential threat, organisms made minor modifications in their enzyme systems to counter ROS [14,16] (Figure 1). This phenomenon may explain the chemical similarity between peroxide and H_2_S or RSS and the interaction between their enzyme systems.

Wang et al. first coined the term ‘gasotransmitters’ to denote gaseous molecules that are produced and regulated endogenously, permeate through lipid membranes, and affect multitudes of cellular functions [22]. Over the years, important cardiopulmonary functions were attributed to NO and CO signaling. Only in the last two decades has H_2_S, which used to be known for its pungent and poisonous nature, been reinvented as the third important gasotransmitter. Since then, H_2_S, RSS, and their downstream signaling pathways have been studied in many tissues [23,24]. H_2_S has been shown to have cytoprotective, anti-inflammatory, and redox-regulatory effects in various tissues and cell types and its perturbation has been associated with multiple disease phenotypes [25,26,27,28]. Consequently, there has been a heightened interest in H_2_S and its signaling in recent years.

## 3. Sulfur Homeostasis and Metabolism

H_2_S levels are dynamically controlled within a very narrow range that represents the aggregate of its rate of formation and degradation [29]. Intracellular levels can range from <1 µM to >100 µM with the steady-state concentration in the nanomolar range [30]. H_2_S easily dissolves in water and dissociates into H^+^, HS^−^, and elemental sulfur. Endogenous H_2_S production can occur through enzymatic or nonenzymatic pathways. Enzymatic production is generally considered as the principal source of H_2_S; however, a recent study has shown that nonenzymatic production may be an important source in several different tissues (e.g., lung, brain, gut) [31]. The study also found that the primary substrate for nonenzymatic production of H_2_S is cysteine (Cys). This pathway is, however, understudied and underappreciated. The other nonenzymatic pathway of H_2_S generation involves the reduction of sulfur from a persulfide or polysulfide in the presence of an electron acceptor such as NADPH [32].

### 3.1. H_2_S Biogenesis

**Figure 2:**
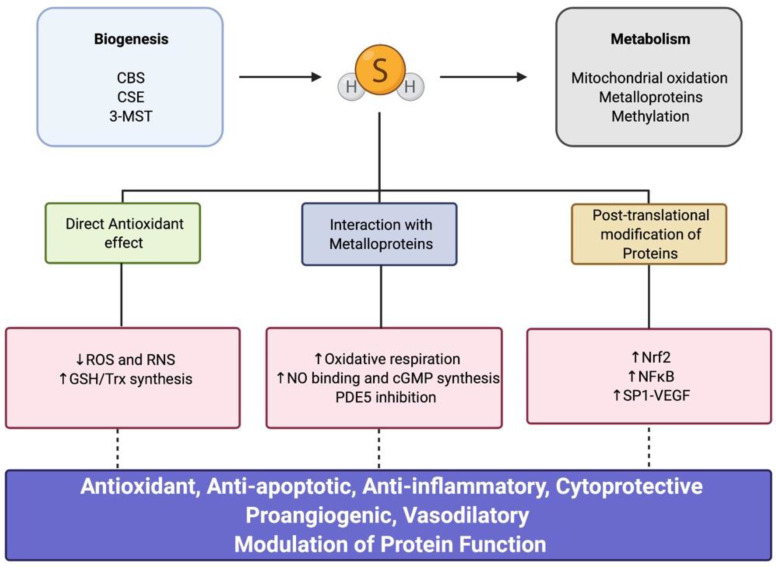
Hydrogen sulfide (H2S) biogenesis, metabolism, and downstream signaling. CBS, cystathionine β-synthase; CSE, cystathionine γ-lyase; 3-MST, 3-mercaptopyruvate sulfurtransferase; ROS, reactive oxygen species; RNS, reactive nitrogen species; GSH, glutathione; Trx, thioredoxin; NO, nitric oxide; cGMP, cyclic guanosine monophosphate; PDE5, phosphodiesterase 5; Nrf2, nuclear factor erythroid 2-related factor; NF-κB, nuclear factor kappa-light-chain-enhancer of activated B cells; SP1, specificity protein 1; VEGF, vascular endothelial growth factor.

Enzymatic production of H_2_S is controlled through the transsulfuration pathway [33]. Three key enzymes are involved in the production of H_2_S: cystathionine β-synthase (CBS), cystathionine γ-lyase (CSE), and 3-mercaptopyruvate sulfurtransferase (3-MST) (Figure 2). The expression of these enzymes differs depending on the tissue and cell type. Two of these enzymes, CBS and CSE, mainly reside in the cytosol, while 3-MST localizes both in cytosolic and mitochondrial compartments [34,35].

CBS serves as a gatekeeper between the methionine cycle and the transsulfuration pathway by catalyzing the first irreversible reaction from homocysteine to cystathionine, thus committing sulfur towards cysteine production and metabolism [36]. The reaction generates H_2_S as a byproduct and thus CBS is an important regulator of H_2_S biosynthesis. CBS is the most common enzyme deficiency seen in the autosomal recessive disorder hereditary homocystinuria [37]. CBS can also serve as a redox sensor and interact with CO and NO, making it an important molecule in the intersection of the three gaseous signaling systems [38]. The enzyme is constitutively expressed in tissues and its expression can be modified through several post-translational modifications, such as sumoylation, glutathionylation, and phosphorylation [33].

CSE, the second enzyme in the transsulfuration pathway, primarily catalyzes the conversion of cystathionine to cysteine [7,39]. This reaction serves as an important source for the amino acid cysteine, which is essential for the glutathione pool in various tissues [40]. CSE is not expressed constitutively and its expression can be induced by a range of oxidative stressors [33]. The promoter site for CSE has a binding site for nuclear factor erythroid 2-related factor (Nrf2), which is the master regulator for oxidative stress [41,42]. CSE can also undergo post-translational modifications similar to CBS, resulting in changes to its localization (e.g., nuclear localization) and function; however, the predominant control occurs at the transcriptional level [43]. Both CBS and CSE can catabolize cysteine to produce H_2_S as a byproduct.

3-MST is located both in the cytoplasmic and mitochondrial compartments of most mammalian tissues [44]. It is a sulfurtransferase enzyme that contains several redox-sensitive cysteine residues, which serve as important regulators of its function, and is unlike CBS and CSE, which are primarily regulated at the translational or post-translational level [45]. 3-MST is also an important H_2_S-generating enzyme. In the 3-MST-related pathway of H_2_S production, cysteine is initially converted through the enzyme cysteine aminotransferase to 3-mercaptopyruvate (3-MP), which acts as a substrate for 3-MST to produce an enzyme-bound persulfide, which in turn can give rise to H_2_S [46].

### 3.2. H_2_S Metabolism

H_2_S metabolism is tightly controlled through the mitochondrial sulfide oxidation pathway, which acts as a bridge to the electron transport chain (ETC) at the level of complex III on the inner mitochondrial membrane [47]. H_2_S toxicity leads to uncoupling of the ETC through inhibition of cytochrome c oxidase (complex IV) [48]. Thus, H_2_S concentration inside the cell is maintained in a narrow range through an intricate balance between its biogenesis and degradation. H_2_S is initially oxidized to a persulfide inside the mitochondria by the enzyme sulfide quinone oxidoreductase (SQR) [47]. The persulfide is further oxidized by the enzyme persulfide dioxygenase (PDO) or ethylmalonic encephalopathy 1 protein (ETHE1) to further produce sulfite. The sulfite is then oxidized by rhodanese or sulfite oxidase in a tissue-specific manner to produce either thiosulfate (lung), sulfate (liver), or a mixture of thiosulfate and sulfate (kidney) [49,50]. Electrons produced through these reactions are transferred to complex III of the ETC through ubiquinone. H_2_S metabolism can thus drive oxidative phosphorylation and ATP synthesis in the mitochondria.

In the extracellular and vascular space, the interaction of H_2_S with metalloproteins, such as methemoglobin and metmyoglobin, can result in its clearance from the circulation by forming sulfheme products [51,52]. This pathway of H_2_S metabolism also marks an intersection between other gasotransmitter signaling pathways [53]. Another alternative mechanism for H_2_S clearance is methylation through the enzyme thiol-s-methyltransferase and mostly occurs in the cytoplasm; however, it is not as important as the other two mechanisms [54].

## 4. Mechanisms of H_2_S Signaling

Over the years, several studies have characterized the versatile role of H_2_S as an important modulator of redox signaling pathways, having antiapoptotic and cytoprotective properties and being a regulator of the inflammatory response in several tissues and cell types [23,55]. Three main underlying mechanisms have been described for H_2_S: (a) direct antioxidant effect, (b) interaction with metalloproteins, and (c) post-translational modification of proteins (Figure 2). In recent years, additional novel pathways of H_2_S and its downstream signaling have been described; however, they are beyond the scope of this review [23].

### 4.1. Direct Antioxidant Effect

Numerous studies have demonstrated that H_2_S can effectively interact with ROS and reactive nitrogen species (RNS) [56]. In fact, in many situations, H_2_S can scavenge reactive intermediates more efficiently than other antioxidants such as cysteine or GSH. H_2_S is particularly effective in juxtacrine signaling mechanisms given its gaseous state, allowing it to freely diffuse through lipid bilayers [57]. H_2_S also plays a role in GSH synthesis through positive feedback in several tissues (e.g., lung, brain, liver, and kidney) to protect against oxidative stress [58,59]. Furthermore, H_2_S can also increase the intracellular production of thioredoxin 1 (Trx1), which protects cells from oxidative injury and promotes peroxidase-dependent detoxification of hydrogen peroxide [60]. Nicholson et al. showed that H_2_S demonstrated cardioprotective effects through the upregulation of Trx1 [61]. H_2_S is a weak reductant and can scavenge free radicals such as superoxide (ROS) or peroxynitrite (RNS) directly [62]. Additionally, H_2_S can dissociate to form HS^•−^, which is a powerful reductant and can scavenge ROS/RNS effectively [63,64]. It is, however, worth mentioning that even though H_2_S can scavenge free radicals effectively, its low nanomolar concentration inside the cells does not compare to the impact of classical antioxidants such as GSH, which are present in micromolar concentrations [30].

### 4.2. Interaction with Metalloproteins

H_2_S can interact with metal centers of metalloproteins, resulting in a reduction or covalent modification [65]. An important example of H_2_S–metalloprotein interaction is in the ETC with the enzyme cytochrome c oxidase (complex IV), which is the final electron acceptor. Cytochrome c oxidase uses electrons provided through cytochrome c to reduce oxygen into water [66]. The enzyme contains two copper and two iron centers. CO and NO can inhibit the enzyme through interaction with its metal centers, thus making them a catalytic site for crosstalk between these gasotransmitter pathways. H_2_S, on the other hand, has a biphasic dose-dependent interaction with cytochrome c oxidase such that at low concentrations (~3 µM), it promotes cellular respiration while irreversibly inhibiting it at higher levels (30–100 µM) [67]. H_2_S can also interact with cytochrome c at a low concentration, resulting in its reduction and subsequent formation of RSS, which can drive further redox reactions downstream [68]. The activity of soluble guanylate cyclase, which is essential for NO signaling, can be modified by H_2_S [69]. The heme iron in soluble guanylate cyclase is reduced by H_2_S, thus promoting NO binding and subsequent cyclic guanosine monophosphate (cGMP) synthesis, which is critical for vasodilation. As mentioned above, several other metalloproteins such as methemoglobin, metmyoglobin, and metneuroglobin serve as a reservoir for scavenging excess H_2_S, thus protecting tissues/cells from H_2_S toxicity [51]. Sulfheme products produced have a much lower affinity for oxygen, which prevents their oxidation and may subsequently be protective against the formation of atherosclerosis [70]. Finally, H_2_S can interact with zinc-containing proteins. H_2_S can inhibit androgen receptor activation by interacting with its zinc finger motif [71]. Additionally, H_2_S in low concentrations can inhibit phosphodiesterase 5, which is a zinc-containing enzyme [72]. H_2_S–zinc interaction is, however, understudied.

### 4.3. Post-Translational Modification of Proteins

H_2_S can modify critical cysteine residues on proteins through a process called persulfidation [73]. Persulfidation is a post-translational modification of proteins and can result in the alteration of protein structure and function. There are also low-molecular-weight persulfides (e.g., cysteine persulfide, glutathione persulfide) that serve as intermediate RSS products and demonstrate strong antioxidant and cytoprotective properties [20]. Low-molecular-weight persulfides are found in very low concentrations inside the cell. On the other hand, persulfidation of protein cysteine residues is a relatively common phenomenon in the cellular proteome [74]. Persulfidation is generally driven by the enzymes CBS and CSE and can be repressed by inhibiting these enzymes [75]. Interestingly, persulfidation is closely related to nitrosation, as one of the studies demonstrated that several cysteine targets in the proteome had an overlap of both processes [76]. On numerous occasions, persulfidation and nitrosation of the same cysteine residue exerted different or even opposite effects [77]. Several important proteins related to redox homeostasis of the cell are persulfidated, resulting in modulation of their function. We will discuss a few of them that are potentially important in the context of neonatal lung redox homeostasis. NF-κB (nuclear factor kappa-light-chain-enhancer of activated B cells), which is an essential transcription factor for antiapoptotic activity, is activated by H_2_S through persulfidation of its p65 subunit at Cys^38^ [78]. Another transcription factor known as SP1 (specificity protein 1), which is a regulator of endothelial function, is persulfidated at multiple cysteine residues, which in turn modulates vascular endothelial growth factor (VEGF) and neuropilin-1 expression [79]. Finally, Nrf2, which is the master regulator of antioxidant response inside the cell, is also modulated by H_2_S through persulfidation [42]. Kelch-like ECH-associated protein 1 (Keap1) normally binds and retains Nrf2 in the cytoplasm, rendering it inactive. Recent studies have shown that H_2_S can persulfidate Keap1 at Cys^151^, resulting in the release of Nrf2 and causing its nuclear localization [80]. After Nrf2 enters the nucleus, it can activate antioxidant response elements (ARE) in promoters of genes, directly supporting activities of GSH and Trx superfamilies and heme oxygenase 1. Interestingly, recent studies suggest that this pathway of Nrf2 activation may be the primary underlying mechanism for upregulation of GSH and Trx by H_2_S rather than direct positive feedback, as was previously described [73].

## 5. Current State of Antioxidant Therapy in Oxidative Neonatal Respiratory Disease

Bronchopulmonary dysplasia (BPD), characterized by an arrest in alveolar and vascular development, is the most common comorbidity in preterm infants [81], affecting 30–60% of infants born very prematurely [82]. Even preterm infants without the diagnoses of BPD endure long-term and persistent pulmonary dysfunction in the form of repeated respiratory infections, recurrent wheezing disorders, and airway hyperreactivity [83,84].

### 5.1. Treatment Modalities

Studies observing the increased concentration of ROS in premature infants who develop BPD [85] and genetic association studies linking polymorphisms of key redox enzymes and outcomes [86], in addition to experimental animal models of BPD, collectively demonstrate the key role of ROS as modulators of lung disease and have triggered several attempts to ameliorate lung injury with the use of antioxidants [87,88]. Some therapies were aimed to augment nonenzymatic and enzymatic antioxidants as well as the use of exogenous administration of vitamins and micronutrients to scavenge ROS [89,90,91]. One of the most studied is N-acetylcysteine (NAC), which acts as a Cys precursor and thiol donor in the glutathione (GSH) system and has been used with success in many other diseases such as chronic bronchitis and chronic obstructive pulmonary disease (COPD) [92,93]. In preterm infants, a double-blind placebo-controlled trial showed no impact on the severity or incidence of BPD nor improved lung function when administered intravenously [90]. Similarly, and despite strong preclinical data, agents such as superoxide dismutase (SOD), vitamin E, and others have been administered to babies, with perhaps only vitamin A showing the most positive but still modest result of decreasing oxygen requirement at 36 weeks [89].

### 5.2. Possible Reasons for the Antioxidant Therapy Failures

Despite mixed and somewhat underwhelming results of clinical trials, much can be learned from them, and in the light of recent laboratory research findings, several speculations can be made to design successful future interventions. First, it is well established that the first week of life is when much of the lung injury occurs in the preterm infant [94,95,96,97]. The brisk shift from the intrauterine to the rather hostile but life-sustaining NICU (Neonatal Intensive Care Unit) environment leads to an immediate and long-lasting oxidative-stress-induced injury. Despite this observation, most interventions aimed to prevent lung disease began many hours up to several days after birth, missing this key treatment window. The experience with prenatal steroids, as perhaps the single and most effective way to prevent BPD [98], indicates that we should design future interventions for even before the infant is born and focus research efforts in understanding maternal–fetal effects of new or known agents.

Another important characteristic of all preterm infants is the absence of the last trimester maternal–fetal nutritional transfer. This period, which preterm-born infants lack, is fundamental for building stores and supplies of many nutrients that are pivotal for antioxidant function. For example, selenium (Se) is an essential trace element that serves as a substrate for selenoproteins, including oxidoreductases such as glutathione peroxidases (GPx) and thioredoxin reductases (TrxR) [99]. Very preterm infants, those most likely develop BPD, are thought to be Se-deficient. Se supplementation was not enough to prevent BPD [100], but coadministration of Se with an additional agent may be an attractive alternative.

A third aspect to consider is the possibility of agents that can upregulate the infants’ endogenous response rather than administrating an exogenous antioxidant. Exogenous antioxidants lack target specificity and have unknown bioavailability, making their effects difficult to predict or interpret. Aiming therapies toward cellular mechanisms that could enhance enzymatic systems and ‘prepare’ the preterm infant to the outside world must be considered. Nrf2 is one of those potential targets that has been explored in other etiologies but not in BPD [101]. As described above, Nrf2 induces antioxidant response genes via the activation of antioxidant response element (ARE) in the promoter/enhancer regions of target genes. Nrf2 plays a crucial role in executing the cellular response to oxidative injury and may provide an opportunity to directly prime endogenous antioxidant systems.

Nonetheless, the apparent failure of antioxidant therapy trials has also prompted the need to explore novel antioxidant mechanisms. We believe that exploring H_2_S and its downstream signaling pathway can fill a critical gap and uncover promising future targets for the therapy of neonatal respiratory diseases.

## 6. H_2_S in the Developing Lung and Neonatal Respiratory Diseases

**Figure 3:**
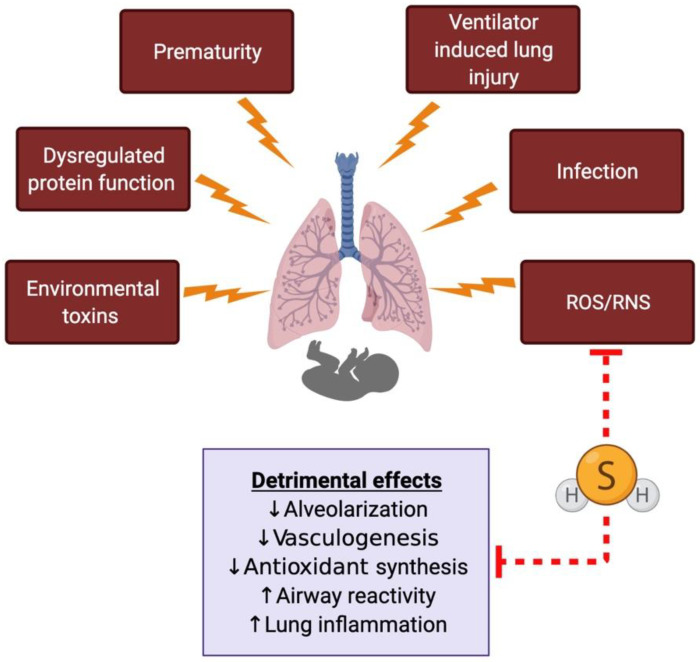
The proposed role of hydrogen sulfide (H_2_S) in the developing lung and neonatal respiratory diseases. The developing neonatal lung is subjected to different kinds of insults, leading to oxidative stress-related damage. H_2_S can directly scavenge free radicals and has been shown to prevent or reverse some of the detrimental effects in animal models of neonatal respiratory diseases. ROS, reactive oxygen species; RNS, reactive nitrogen species.

Most of the studies with H_2_S in the lung have been done using models of adult lung diseases; however, recent studies have demonstrated an emerging role of H_2_S and sulfide signaling in the developing lung [28] (Figure 3). Research across many species has demonstrated the presence of important H_2_S enzymes in different lung tissue compartments and the lung vasculature [8,102].

Recently, Bartman et al. showed that the H_2_S machinery including the metabolizing enzymes (SQR, ETHE1) are present and functional in the human fetal airway smooth muscle cells [10]. The synthesizing enzymes (CBS and CSE) were, however, expressed in a lower amount in the fetal airway when compared to adults, suggesting decreased H_2_S production in the preterm airways. Decreased H_2_S would limit the ability of the preterm airway and lung to counter oxidative stress from hyperoxia- or ventilator-induced lung injury. Indeed, they found that the supplemental oxygen altered the expression of enzymes associated with H_2_S biogenesis and metabolism, which resulted in further blunted H_2_S production. Additionally, by using external H_2_S donors (e.g., NaHS or sodium hydrosulfide, GYY4137 or morpholin-4-ium 4-methoxyphenyl(morpholino) phosphinodithioate), it was possible to reverse the detrimental effect of oxygen exposure on airway constriction response (i.e., airway reactivity), resulting in diminished intracellular calcium response to bronchoconstrictor agonists.

In an earlier study, Madurga et al. showed in a murine model of BPD that exogenous administration of H_2_S improved alveolarization and vascular growth following hyperoxia exposure [9]. In a later study, the same group showed that CBS is mainly expressed in the lung/airway epithelial cells and pulmonary vessels, whereas CSE is initially expressed predominantly in the lung parenchyma, and eventually its expression is upregulated in the airway [8]. Furthermore, CBS and CSE were found to play an important role in vasculogenesis during normal alveolar development. This is an intriguing finding and underscores the fact that the H_2_S machinery plays an important role in lung development in utero, which happens to be a relatively hypoxic environment [103]. Extrapolating from what we know from human fetal development and evolutionary remnants, this finding reiterates the evolutionary importance of H_2_S and its downstream signaling, which once used to be the predominant energy source in an environment that was severely hypoxic [14].

H_2_S production is distinguished from the production of the other two gasotransmitters CO and NO since oxygen is not essential. H_2_S consumption, on the other hand, requires oxygen and is related to oxidative phosphorylation, which means H_2_S and oxygen concentrations in a system tend to be inversely proportional [104]. This was shown in a study with rat lungs in a hypoxic environment where the tissue concentration of H_2_S decreased swiftly as oxygen was added [105]. In this regard, H_2_S indirectly acts as an oxygen sensor in the tissues. One school of thought is that H_2_S is produced constitutively in tissues and its metabolism, specifically H_2_S clearance, depends on environmental oxygen tension [104]. However, it may not be that simple, as H_2_S-generating enzymes, especially CSE, are highly inducible and respond to various stimuli including oxidative stress [55]. This means normoxia (or hyperoxia) may initially upregulate H_2_S production, but given the rapid clearance in the presence of increased oxygen, the H_2_S levels in the tissues are still maintained in a low and narrow range. This is supported by a study that measured the urinary metabolite of H_2_S (thiosulfate) in term and preterm human infants as a measure of H_2_S turnover rate and found that the highest H_2_S turnover rate was seen in very preterm infants [106]. Given what is known about the relationship between H_2_S and oxygen, it is reasonable to assume that the H_2_S machinery is highly active in fetal life with a high concentration of H_2_S, which rapidly falls after birth following exposure to higher oxygen tension.

Several important mediators of normal lung development and angiogenesis—such as hypoxia-inducible factor-1 alpha (HIF-1α) and vascular endothelial growth factor (VEGF)—are known to be sensitive to changes in oxygen tension [107]. Interestingly, both these mediators are directly or indirectly modulated through H_2_S and its downstream signaling. An elegant study published recently demonstrated that CBS modulates HIF-1α stability through persulfidation of its inhibitor [108]. Further, H_2_S can stabilize the transcription factor SP1 through persulfidation, which in turn modulates the expression of VEGF receptor 2 [79]. As described above, H_2_S can regulate Nrf2 activation and its downstream antioxidant response inside the cell [42,80]. Given the emerging roles of Nrf2 and associated GSH and Trx superfamilies in BPD [101,109,110,111,112], it would be interesting to explore the crosstalk between H_2_S and the Nrf2 signaling pathways.

H_2_S has also been shown to have protective effects on the lung in studies using ventilator-induced lung injury models [113]. Exogenous administration of an H_2_S donor was shown to be protective to both mouse and rat lungs when used prophylactically or during ventilation [114,115]. In animal models of adult COPD using environmental toxin exposure (cigarette smoke), H_2_S was shown to be protective (Figure 3) [116,117]. H_2_S and sulfide signaling has been explored in numerous animal models of airway hyperreactivity and asthma [118,119,120]. In population-based studies, H_2_S levels in different body fluids (sputum, serum) correlated with the degree of airway inflammation in both children and adults [121,122]. Additionally, exhaled H_2_S is a marker for airway inflammation in asthmatic patients [123]. All these studies suggest a role of H_2_S as a biomarker for the severity of respiratory diseases.

Finally, loss of the H_2_S-generating enzyme CSE in an airway epithelial cell model for the respiratory syncytial virus (RSV) increased the severity of the infection, augmented inflammatory damage, and worsened airway reactivity [124,125]. H_2_S has also been touted as a prophylactic and/or therapeutic agent against the novel SARS-CoV-2 and a potential biomarker for COVID-19 disease severity [126,127,128].

## 7. Future Direction

Preterm infants deficient in hepatic CSE activity are already prone to impaired transsulfuration [129,130], and human fetal airway smooth muscle cells have decreased transcript and protein expression of H_2_S-synthesizing (CBS, CSE) and -metabolizing (SQRDL, ETHE1) enzymes compared to adult cells [10]. H_2_S production was further blunted in fetal airway smooth muscle cells by 40% hyperoxia after two days in culture [10]. This raises a strong interest in therapeutic opportunities targeting H_2_S for oxidative neonatal respiratory disease, along with data supporting that H_2_S synthesis is required for proper programming of perinatal alveolarization [8] and that H_2_S donors attenuate hyperoxic lung injury in neonatal rodents [9,131]. Therapeutic targeting of H_2_S is possible via donors, amplification of endogenous H_2_S synthesis, or direct delivery of the gasotransmitter to the lung via the trachea. A recent review published on this subject paints a similar picture regarding an experimental approach to neonatal respiratory diseases using H_2_S [132]. Sulfide signaling is an evolutionary conserved pathway, which presents an exciting prospect to uncover promising targets for future therapies. H_2_S machinery is endogenously present and active, which may suggest a lower probability of detrimental off-target effects with optimal bioavailability. However, upon reflection on lessons learned from failed antioxidant therapies, there are still several key issues that require a greater depth of investigation before considering H_2_S-targeted therapeutic opportunities for oxidative neonatal respiratory disease: cell-specific physiologies, identification of molecular networks modified through persulfidation of protein cysteine thiols, intersection with other redox-dedicated pathways, dynamic changes as a function of developmental timing, and influences of prematurity and/or hyperoxic injury on the aforementioned molecular and cellular events.

Systemic targeting of H_2_S is a common limitation for highly encouraging studies demonstrating preclinical efficacy of H_2_S donors in experimental hyperoxic rodent models of BPD [9,133] and CBS and CSE promotion of perinatal alveolarization using whole-body knockout mice [8]. H_2_S biology is complex with cell-specific and context-dependent effects. Similar spatial expression patterns of CBS and CSE in airway epithelium and vessel walls (colocalization with smooth muscle actin) were detected in neonatal mouse and human lung samples [8,10]. This is in relative agreement with single-cell RNA sequencing data from LungMAP demonstrating murine pulmonary epithelial, endothelial, and fibroblast expression of CBS, CSE, and 3-MST across late embryonic and neonatal ages [133]. The cell-specific influence of H_2_S biosynthesis enzymes in the contexts of mammalian lung development and experimental models of oxidative neonatal respiratory disease require further investigation, since resulting data could be critical for determination of preferred therapeutic strategy.

Furthermore, taking a systems-level approach to H_2_S biology is likely to reveal ubiquitous and cell-specific functions of H_2_S. First, H_2_S biosynthesis and metabolism enzymes could be differentially regulated in a cell-specific manner. CBS and CSE activities are influenced by several different post-translational modifications [33]. Akt increased catalytic activity of CSE through direct binding and phosphorylation in liver sinusoidal cells [134]. Interestingly, treatment of neonatal mice treated with an H_2_S donor during 85% oxygen exposure for the first ten days of life had increased Akt activation (phosphorylation) in the whole lung [9]. Although H_2_S donors caused similar Akt activation in mouse primary alveolar type II cells and MLE-12 cells, H_2_S-induced Akt activation was oxygen-dependent in vivo. While it is unknown if Akt activation augments endogenous H_2_S synthesis through CSE phosphorylation, this further underscores the need to investigate cell-specific and context-dependent H_2_S biology and sulfide signaling. Second, it is likely that H_2_S-dependent protein cysteine thiol persulfidation targets different molecular networks and/or has different kinetics across various cell types. It is possible to map H_2_S protein networks by identifying S-sulfhydration of protein cysteine thiols using a maleimide assay [135]. This biochemical approach would give better mechanistic context to understanding H_2_S-mediated effects and could identify additional novel therapeutic targets downstream of H_2_S. Lastly, it is important to consider how H_2_S-depenedent processes intersect with other redox-dedicated pathways to coordinate cellular and physiological outcomes. Although data are conflicting, it is clear that H_2_S influences NO and GSH pathways, which can in turn also regulate H_2_S processes [136].

One powerful approach that should be employed to delineate cell-specific H_2_S effects is to ablate or modify genes encoding for H_2_S-synthesizing enzymes using Cre-mediated recombination in the mouse. There is a plethora of mouse strains that have been engineered to express Cre recombinase in lung using epithelial, endothelial, fibroblast, and smooth muscle-specific promoters [137]. This includes a subset of tetracycline- and tamoxifen-inducible systems for temporal control of Cre recombinase. Conditional control of Cre-dependent genetic ablation or modification of H_2_S-synthesizing enzymes initiated during precise embryonic and perinatal developmental windows, as well as during hyperoxic exposure, would allow for temporal mapping of systems-level H_2_S-dependent biochemical and molecular processes. However, there are several limitations and caveats regarding spatial expression of Cre recombinase, recombination efficiency, and off-target effects. One last limitation, specifically influencing generation of double- and triple-transgenic mouse lines, is strain-dependent redox responses. Inbred mouse strains have differential responses to hyperoxic lung injury [138,139], and it is possible that hyperoxic pathologies in the widely popular C57Bl/6J strain are influenced by a mutation impairing mitochondrial NADPH synthesis [140]. NADPH provides reducing potential for both glutathione and thioredoxin antioxidant systems. Although there is optimism and enthusiasm for H_2_S-mediated therapeutic opportunities in neonatal oxidative respiratory disease, additional depth of knowledge on H_2_S biology and factors influencing H_2_S activities in a cell-specific context is needed, otherwise future clinical trials could meet a similar fate of failed antioxidant therapies.

## 8. Conclusions

H_2_S and its downstream signaling pathway are emerging as novel and important players in lung development. Recent studies have demonstrated that exploring H_2_S signaling pathways can uncover promising therapeutic targets for acute and chronic respiratory diseases associated with prematurity. While exogenous H_2_S donors have primarily been used to treat or prevent lung injury, it is not completely clear how the endogenous H_2_S pathway and enzymes are regulated. Future studies should focus on exploring the regulation and signaling of endogenous H_2_S machinery in the context of oxidative neonatal respiratory disease. Additionally, sulfide signaling, being an ancient pathway, also carries importance from an evolutionary perspective. Evidence, although limited, does suggest highly active H_2_S machinery in premature babies, much like in the Archean era, when H_2_S served as the major energy source for the extant organisms. More innovative studies using novel in vitro models such as embryonic lung explant cultures and lung organoid cultures are needed to gain a clear understanding of this intriguing phenomenon.

## Data Availability

Not applicable.
